# Anti-inflammatory effect and mechanism of stytontriterpene D on RAW264.7 cells and zebrafish

**DOI:** 10.3389/fphar.2025.1559022

**Published:** 2025-04-28

**Authors:** Chuqin Yu, Gao Qiu, Xiangying Liu, Quanwei Xie, Zonghao Lin, Feng Wang, Lei Cai

**Affiliations:** ^1^ Centre for Drug Research and Development, Guangdong Pharmaceutical University, Guangzhou, China; ^2^ Guangdong Provincial Key Laboratory for Research and Evaluation of Pharmaceutical Preparations, Guangzhou, China; ^3^ Guangdong Engineering and Technology Research Center of Topical Precise Drug Delivery System, Guangdong Pharmaceutical University, Guangzhou, China; ^4^ School of Chinese Materia Medica, Guangdong Pharmaceutical University, Guangzhou, China; ^5^ Guangdong Provincial Biotechnology Research Institute (Guangdong Provincial Laboratory Animals Monitoring Center), Guangzhou, China

**Keywords:** stytontriterpene D, anti-inflammatory, mechanism, RAW 264.7 cell, zebrafish

## Abstract

**Introduction:**

Stytontriterpene D ( STD ) is a compound isolated from dried resin of *Styrax tonkinensis (Pierre) Craib ex Hartw*. In this study, we explored the anti-inflammatory effect of STD *in vitro* and *in vivo* and examined its potential anti-inflammatory mechanism for the first time.

**Methods:**

*In vitro*, we evaluated the toxicity of STD to RAW 264.7 cells using the CCK8 method and detected the reactive oxygen species (ROS) and nitric oxide (NO) contents in cells using diacetyldichlorofluorescein (DCFH-DA) and the Griess method. We detected the levels of interleukin-6 (IL-6), interleukin-1β (IL-1β), tumor necrosis factor-α (TNF-α), inducible nitric oxide synthase (iNOS), interleukin-10 (IL-10), and arginase-1 (ARG1) via enzyme-linked immunosorbent assay and measured the expression of related proteins in the NF-κB pathway via western blotting. The toxicity of STD to AB zebrafish was detected *in vivo*, and the recruitment of neutrophils and macrophages was evaluated in tail cut-induced and copper sulfate-induced zebrafish inflammation models. We used quantitative real-time polymerase chain reaction to study the expression of inflammation-related genes in zebrafish with inflammation induced by copper sulfate.

**Results:**

In lipopolysaccharide (LPS)-induced RAW 264.7 cells, STD decreased IL-6, IL-1β, NO, ROS, and TNF-α production, and increased the expression of IL-10 and ARG1 while also blocking inhibitory κBα (IκBα) phosphorylation and suppressing P65 nuclear translocation. STD also reduced the recruitment of inflammatory cells in zebrafish with inflammation induced by tail cutting and copper sulfate. STD not only reduced the copper sulfate–induced gene expression of zebrafish inflammatory factors, but it also inhibited the mRNA levels of NF-κB p65 and IκBα.

**Conclusion:**

These results demonstrated that STD has an obvious anti-inflammatory effect, and its intrinsic molecular mechanism is possibly caused by inhibiting the NF-κB signaling pathway and regulating the phenotypic changes of M1 and M2 macrophages. Thus, STD may play a potential role in the treatment of inflammatory diseases.

## 1 Introduction

Inflammation comprises a sequence of protective immune responses activated by the host system in response to pathogens, damaged cells, or other foreign bodies. This process is pivotal in eliminating harmful stimuli and initiating healing within the body. The inflammatory cascade is initiated by various cytokines and mediators released from pro-inflammatory cells, such as macrophages (Chen et al., 2017; Kim et al., 2017). While inflammation is beneficial to the body’s physiological function under normal conditions, an excessive or persistent inflammatory response can be detrimental. To some extent, inflammation plays a role in the pathogenesis of various human diseases, ranging from infections and immune-mediated disorders to cardiovascular diseases, diabetes, metabolic syndrome, neurodegenerative diseases, cancer, and aging (Arulselvan et al., 2016; Rea et al., 2018). Clinically, anti-inflammatory drugs are primarily categorized into steroidal and non-steroidal agents, which often carry numerous side effects. Prolonged usage can lead to damage in the gastrointestinal tract, liver, urinary system, nervous system, and beyond. Anti-inflammatory drugs derived from natural sources can compensate for the limitations of currently available medications and maintain a significant position in drug discovery. As an illustration, resveratrol, extracted from grapes, and curcumin, extracted from curcuma, exhibit potent anti-inflammatory properties (Sen and Samanta, 2015; Peng et al., 2021; Gu et al., 2024). Natural products offer rich chemical diversity, less adverse reactions and multi-target mechanisms in treating inflammation. The development of natural anti-inflammatory drugs is an important direction of future pharmaceutical innovation (Ruan et al., 2024; Koeberle and Werz, 2014; Gouda et al., 2023). Consequently, discovering novel anti-inflammatory agents from natural origins and elucidating their mechanisms of action holds immense importance.

Lipopolysaccharide (LPS) is the primary component of the cell wall of Gram-negative bacteria and induces macrophages to transform into M1 phenotype with pro-inflammatory effects through classical pathways, and releases pro-inflammatory mediators and cytokines by activating toll like receptor 4 (TLR4) - mediated nuclear factor-kappa B (NF - κB) signaling pathway (Yang et al., 2016)**.** These mediators include interleukin-6 (IL-6), interleukin-1β (IL-1β), nitric oxide (NO), inducible nitric oxide synthase (iNOS), and tumor necrosis factor-α (TNF-α) and they are a pivotal part of the innate immune response of mammals, taking the roles of pro-apoptotic and cytotoxic mediators (Hankittichai et al., 2020). Consequently, LPS has been implicated in various inflammatory diseases and is commonly utilized to induce inflammatory disease models such as stimulating macrophages. Macrophage polarization can be divided into two types: M1 macrophages and M2 macrophages Under normal circumstances, M1 and M2 are in dynamic equilibrium and can switch to each other in inflammatory reactions. In the early stages of inflammation, M1 macrophages release pro-inflammatory and chemokines to recruit and activate immune cells, but may cause tissue damage. In the recovery stage of inflammation, M1 converts to M2, mainly induced by interleukin-4 (IL-4) or interleukin-10 (IL-10), participating in anti-inflammatory reactions, exerting anti-inflammatory effects, and participating in tissue healing and repairing (Yunna et al., 2020). Therefore, regulating the polarization of M1/M2 macrophages may be an important intervention measure to suppress the occurrence of inflammation. The NF-κB signaling pathway, which can be activated by LPS, is a critical factor in triggering diseases related to inflammation and causing them to progress (Guha and Mackman, 2001). NF-κB is a transcription factor composed of p50 and p65 subunits. Within the typical signaling pathway, this factor is essential in regulating the gene transcription that produces pro-inflammatory mediators and cytokines. Under regular conditions, NF-κB exists as a dimer in the cytoplasm due to its binding with the inhibitory IκB subunit. Upon activation by LPS or other stimuli, NF-κB initiates the phosphorylation and degradation of IκBα, enabling the NF-κB subunit to relocate to the nucleus. Not only does it regulate the response of cells to various cytokines, but it also promotes the production of reactive oxygen species (ROS) and pro-inflammatory cytokines (e.g., IL-1β, IL-6, and TNF-α) (Shih et al., 2015; Cao et al., 2021; Gałgańska et al., 2023). As a result, inflammatory diseases can be treated by preventing the activation of the NF-κB signaling pathway.

Zebrafish not only share a high percentage of genetic sequences with humans, but also have a high functional similarity with them. The nervous system and organ development process of zebrafish are very similar to those of humans, which is why zebrafish have become a popular animal model for studying various human diseases (Postlethwait et al., 2000; Liu et al., 2024). Zebrafish also have an immune system structure similar to that of humans, and as such are considered to be an ideal inflammatory model for finding new anti-inflammatory drugs. Inflammation in zebrafish can be caused by physical, chemical, or biological stimuli. Commonly used inducers for establishing zebrafish inflammation models include LPS, copper sulfate, and tail cutting (Zanandrea et al., 2020).

Benzoinum is derived from the dried resin of *Styrax tonkinensis (Pierre) Craib ex Hartw* and contains various constituents, such as triterpenoids, aromatic compounds, and derivatives such as coniferyl and morinol. This substance is extensively used in clinical settings for its aromatic sputum-clearing properties and is noted for its anti-inflammatory, antioxidant, and cardiovascular protective effects (Zhang et al., 2019). In traditional Chinese medicine, benzoinum is frequently used to manage stroke, and the 2015 edition of the pharmacopoeia of the people’s republic of China highlights its application in stroke treatment (Chen et al., 2020). It is an ingredient in several traditional Chinese formulations for stroke, including suhexiang pills and zhibao dan. Clinical research has demonstrated that Suhexiang pills, which contain benzoinum, are more effective than standard treatments for stroke, underscoring their significance in Chinese medicinal practices (Xie Q. et al., 2021). Some studies have suggested that some components in the dry resin can be used to treat these diseases. Our previous studies aimed to identify the anti-atherosclerotic components of resin. Several previously undiscovered oleanane–lactone triterpenes were isolated, one of which was named stytontriterpene D (STD). Experimental results have demonstrated that STD significantly lowers the levels of acute reactants and inflammatory cytokines, and it can also inhibit the adhesion of THP-1 to human umbilical vein endothelial cells (Jiang et al., 2024). As the development of cardiovascular disease is closely related to inflammation, it is reasonable to believe that these newly isolated components may inhibit the immune response and the resulting inflammation. Triterpenoids are a group of natural compounds found in various plants that have garnered significant interest from researchers for their wide-ranging pharmacological properties, including antitumor, antiviral, antibacterial, anti-inflammatory, and immune-regulating effects. Triterpenoids have become particularly noted for their anti-inflammatory capabilities, and as a result have become a focus of research, especially because of their natural origins and ability to target multiple pathways in inflammation (Fan et al., 2020; Miranda et al., 2022).

We speculate that the newly isolated STD has an anti-inflammatory effect, though the anti-inflammatory effect and mechanism of STD have not yet been examined. Thus, we studied STD’s anti-inflammatory activity *in vivo* and *in vitro* in zebrafish models and RAW 264.7 cells. We utilized LPS-induced RAW 264.7 macrophages *in vitro* to assess the mechanism of STD’s anti-inflammatory effect on macrophages. We also studied STD’s inhibitory effect on ROS, NO, iNOS, and other inflammatory factors (e.g., IL-6, IL-1β, and TNF-α) induced by LPS, and explored the impact of STD on macrophage M1/M2 phenotype transition by measuring the expression of IL-10 and ARG1. We induced inflammation in zebrafish utilizing chemical and physical methods (i.e., copper sulfate and tail cutting) to study the effect of STD, respectively. In addition, we measured macrophage aggregation, neutrophil aggregation, and inflammatory gene expression.

## 2 Materials and methods

### 2.1 Reagents

RAW 264.7 mouse macrophages were purchased from the Shanghai Cell Resource Center at the Chinese Academy of Sciences. We also purchased the following: high-glucose Dulbecco’s modified eagle medium (DMEM; ThermoFisher Scientific, Waltham, MA, United States), penicillin streptomycin double antibody (ThermoFisher Scientific), fetal bovine serum (FBS; Procell, Bethel, CT, United States), phosphate-buffered saline (PBS; Eallbio), universal protein-free cryopreservation solution (Eallbio), dexamethasone (DEX; MeilunBio, Liaoning, China), dimethyl sulfoxide (DMSO; Biosharp, Guangzhou, China), LPS (Biosharp), Cell Counting Kit-8 reagent CCK8 (APExBIO, Houston, TX, United States), NO detection kit (Beyotime, Beijing, China), ROS detection kit (Beyotime), IL-6 enzyme-linked immunosorbent assay (ELISA) detection kit, IL-1β (ELISA) detection kit, TNF-α (ELISA) detection kit (Jiangsu Enzyme-Free Industrial Co., Ltd., Jiangsu, China), iNOS (ELISA) detection kit, IL-10 (ELISA)detection kit, ARG1 (ELISA) detection kit (FANKEW, Jiangsu, China) Tricaine (Tixiai Shanghai Chemical Industry Development Co., Ltd., Shanghai, China), NF-κB p65 antibody (Cell Signaling Technology, Danvers, MA, United States), IκBα antibody (Abcam, Cambridge, United Kingdom), phospho-IκBα (Abcam), β-actin antibody (Bioss, Beijing, China), histone H3 antibody (Abbkine, Atlanta, GA, United States), TRIZOL (Tiangen Biochemical Technology Beijing Co., Ltd., Tiangen, China), chloroform (Guangzhou Chemical Preparation Factory, Guangzhou, China), anhydrous ethanol (Biotech Bioengineering Shanghai Co., Ltd., Shanghai, China), Evo M-MLV RT Master Mix (Hunan Aikerui Bioengineering Co., Ltd., Hunan, China), and SYBR Green Pro Taq Hs Premix (Hunan Aikerui Bioengineering Co., Ltd.).

### 2.2 Extraction and isolation

The dried resin of *S. tonkinensis (Pierre) Craib ex Hartw* was taken (5 kg) and extracted with 40 L 95% ethanol at room temperature three times each for 24 h, and then was volatilized under vacuum conditions. The resulting residue (4.2 kg) was dispersed in warm water and extracted with ethyl acetate to obtain an ethyl acetate soluble fraction (3.8 kg). We subjected this product to silica gel column chromatography with petroleum ether and ethyl acetate (50:1–1:1) as mobile phases for gradient elution. Subsequently, we further separated the product through silica gel column chromatography (petroleum ether-ethyl acetate, 40:1–0:1). The obtained product was then eluted with MCI gel CHP20P column and methanol aqueous solution (10%–100%), after which it was separated by MCI gel CHP20P column chromatography. We eluted the product with a methanol aqueous solution (10%–100%) and then separated it with a medium- and low-pressure octadecylsilyl (ODS) column (aqueous methanol, 50%–100%, 8 mL/min). Purification of the obtained product by repeated semipreparative high-performance liquid chromatography (HPLC; methnaol - H_2_O; 55:45) yielded STD (36.0 mg, tR = 35.1 min) (Jiang et al., 2024).

### 2.3 Cell culture

We used high-glucose DMEM with 10% FBS, 100 U/mL penicillin, and 100 μg/mL streptomycin to culture the RAW 264.7 cells at 37°C in a 5% carbon dioxide (CO_2_) incubator.

### 2.4 Cell viability assay

We used a 96-well plate at a density of 2 × 10^4^ cells per well to incubate the RAW 264.7 cells overnight. The cells were treated for 24 h at 37°C utilizing different amounts of stytontriterpene D (6.6, 13.2, 26.5, 53.0 and 106.0 μM). After incubation, we removed the supernatant and added 100 μL of medium with 10% CCK8 to each well. The cells were subsequently incubated for 0.5 h at 37°C. We used a microplate reader to determine the absorbance value at 450 nm. After determining the safe concentration of STD, we conducted a safety experiment on LPS-induced macrophages undergoing STD treatment. Specifically, we aimed to ascertain the survival rate of LPS-induced RAW 264.7 cells during the STD treatment process. The experimental procedure involved several steps: First, we pretreated cells in each well plate with varying concentrations of STD for 1 hour. Next, we added LPS to each well containing the drug and incubated them together for 24 h fter incubation, we removed the supernatant and added 100 μL of medium with 10% CCK8 to each well. The cells were subsequently incubated for 0.5 h at 37°C. We used a microplate reader to determine the absorbance value at 450 nm.

### 2.5 Measurement of ROS level

We used a 12-well plate at a density of 2 × 10^5^ cells per well to incubate the RAW 264.7 cells overnight. The experiment was divided into blank control group (complete medium), LPS group (1 μg/mL), DEX positive control group (30.6 μM) and STD group (6.6 μM, 13.2 μM and 26.5 μM). Following the experimental grouping scheme, cells in each well were treated for 1 h with either STD medium, DEX medium, or complete medium at varying concentrations. Following this pretreatment, all groups except the blank control group were added with LPS and continued to stimulate for 24 h, respectively. The cells underwent three washes with PBS buffer, followed by the addition of 10 μM DCFH-DA. The cells were incubated without light at 37°C for 20 min, after which the PBS buffer was utilized to wash the cells three times. We used an inverted fluorescence microscope to visualize the cells for imaging and used ImageJ software to measure and analyze the fluorescence intensity.

### 2.6 Measurement of NO level

We used a 12-well plate at a density of 2 × 10^5^ cells per well to plate, group, and culture the RAW 264.7 cells for 24 h, using the same method described in Section 2.5. An oscillating instrument was employed to mix the culture supernatant with Griess reagents I and II. Then, the sample was incubated for 10 min at room temperature. Following incubation, we took measurements of the absorbance at 560 nm to determine how much of the NO was concentrated in the supernatant.

### 2.7 Enzyme-linked immunosorbent assay

We used a 12-well plate at a density of 2 × 10^5^ cells per well to plate, group, and culture the RAW 264.7 cells for 24 h, using the same method described in Section 2.5. Each group’s supernatant was subsequently collected, and an enzyme-linked immunosorbent assay (ELISA) kit was used to detect the levels of IL-1β, IL-6, TNF-α, iNOS, IL-10, and ARG1 in each group, following the manufacturer’s instructions.

### 2.8 Western blot

We used a 12-well plate at a density of 2 × 10^5^ cells per well to plate, group, and culture the RAW 264.7 cells for 24 h, using the same method described in Section 2.5. PBS was used to wash the cells three times, and radio immunoprecipitation assay lysis solution (with 1% protease inhibitor) was utilized to extract the total protein. We then used a nuclear protein and cytoplasmic protein extraction kit to extract the proteins and used a BCA total protein quantitative kit to detect the protein concentration in each sample and to subject the protein to sodium dodecyl sulfate-polyacrylamide gel electrophoresis. The target protein was then transferred to the polyvinylidene fluoride (PVDF) membrane, after which tris-buffered saline with tween 20 (TBST) with 5% skim milk was used to shock and block the sample for 30 min. The primary antibodies P-IκBα, IκBα, and NF-κB p65, diluted a ratio of 1:500 in TBST with 5% skim milk, were used to incubate the membrane at 4°C overnight. The membrane was then washed with TBST buffer three times at room temperature and treated with secondary antibodies. We diluted the sample for 30 min to 1:3,000 in TBST at room temperature and then washed the sample three more times with TBST at room temperature. We then exposed the PVDF membrane to enhanced chemiluminescence reagent and developed and used ImageJ software to quantify the band intensity.

### 2.9 Animal care ethics

Male and female adult wild zebrafish (AB type) were purchased from China Zebrafish Resource Center and the research was approved by the Institutional Animal Care and Use Committee (IACUC) of Guangdong Provincial Biotechnology Research Institute (Guangdong Provincial Laboratory Animals Monitoring Center) (protocol code A-IACUC2023106) and was conducted in accordance with the internationally accepted principles for laboratory animal use and care.

### 2.10 Zebrafish maintenance and embryo collection

We kept the zebrafish in a recirculating aquaculture setup with water temperatures maintained at (28.5 ± 1.0)°C. The environment had a pH of 7.0–7.5 and followed a consistent light/dark cycle of 14 h of light and 10 h of darkness. The evening before spawning, male and female zebrafish were placed in mating boxes at a 1:1 ratio, separated by a divider. Upon the onset of light the next morning, we moved the divider, allowing the fish to mate and spawn. Embryos collected 0–2 h post-fertilization (hpf) were rinsed three times with aquaculture water and transferred to a Petri dish. We removed the unfertilized embryos under a stereomicroscope before conducting further experiments with the zebrafish.

### 2.11 Toxicity of STD in zebrafish

We assessed stytontriterpene D toxicity by measuring the survival rate of zebrafish embryos. Specifically, at 6 hpf, the embryos were randomly distributed into a 24-well plate (n = 30 per well) and exposed to different concentrations of STD (6.6, 13.2, 26.5, 53.0 and 106.0 μM) for a duration of 96 hpf, extending until 4 days post-fertilization (dpf). We conducted daily observations under a stereomicroscope to assess the survival rate of the zebrafish embryos.

### 2.12 Tail transection–induced inflammatory model in zebrafish

#### 2.12.1 Effect of STD on neutrophil aggregation in the tail of tail-transection zebrafish (sudan black staining)

The 3-dpf zebrafish were placed in a six-well plate with 10 tails per well. Fish from each group, except for the blank group, were anesthetized using tricaine, and their caudal fins were cut off. Then we added DEX (51.0 μM) and various amounts of stytontriterpene D (13.2, 26.5, and 53.0 μM) and incubated the samples for 4 h. The solution was discarded, fixed overnight with 4% PFA, and washed with PBST. Sudan black staining solution was added and incubated in the dark for 25 min. We rinsed the samples with 70% methanol until the rinse solution was clear. We examined the neutrophil aggregation in the caudal fins of the fish under a stereomicroscope, and we recorded and enumerated the number of neutrophils. The count was done within 100 μm of the site of the incision.

#### 2.12.2 Effects of STD on macrophage aggregation in the tail of tail-transection zebrafish (neutral red staining)

The 3-dpf zebrafish were placed in a six-well plate with 10 tails per well. Fish from each group, except for the blank group, were anesthetized using tricaine, and their caudal fins were cut off. Then we added the DEX (51.0 μM) solution containing neutral red (NR) dye solution and the stytontriterpene D solution containing various amounts of NR dye solution (13.2, 26.5, and 53.0 μM) and incubated the sample for 6 h in the dark. The attached dye solution was washed with aquaculture water, and the juvenile fish were anesthetized with 0.02% tricaine solution. The aggregation of macrophages in the caudal fin of the juvenile fish was observed under a stereomicroscope, photographed and recorded, and the number was counted. The counting range was within 100 μm of the site of the incision.

### 2.13 Copper sulfate–induced inflammatory model in zebrafish

#### 2.13.1 Effect of STD on copper sulfate–induced neutrophil aggregation in zebrafish (Sudan black staining)

The 3-dpf zebrafish were taken and placed in six-well plates with 10 tails per well. Various amounts of STD (13.2, 26.5, and 53.0 μM) and DEX (51.0 μM) were added to each group, except for the blank group, after which they were incubated for 4 h. The samples included 10.0 μM copper sulfate. The solution was discarded, fixed with 4% PFA overnight, and then washed with PBST. Next we added sudan black staining solution and incubated the samples for 20 min without light. The sample was washed with 70% methanol until the washing solution became colorless. The staining of neutrophils in zebrafish lateral line was observed under a stereomicroscope, photographed and recorded, and the number was counted.

#### 2.13.2 Effect of STD on copper sulfate–induced macrophage aggregation in zebrafish (neutral red staining)

The 3-dpf zebrafish were taken and placed in six-well plates with 10 tails per well. Various amounts of STD (13.2, 26.5, and 53.0 μM) and DEX (51.0 μM) were added to each group, except for the blank group, all of which contained 10.0 μM copper sulfate and NR dye solution at different concentrations, respectively, and incubated in the dark for 6 h. The attached dye solution was washed with aquaculture water, and the juvenile fish was anesthetized with 0.02% tricaine solution. The staining of zebrafish lateral line macrophages was observed under a stereomicroscope, photographed and recorded, and the number was counted.

### 2.14 Quantitative RT-PCR and RNA extraction

The SYBR Green Pro Taq Hs Premix was utilized on a QuantStudioTM 5 RT-PCR system to conduct quantitative real-time polymerase chain reaction (qRT-PCR). The PCR cycle included denaturing for 30 s at 95°C, and 40 cycles of denaturing for 5 s at 95°C, annealing for 30 s at 60°C, and extension for 30 s at 72°C. We conducted a dissociation curve analysis with steps for 15 s at 95°C, 1 min at 60°C, 15 s at 95°C, and 15 s at 60°C. We used the 2^−ΔΔCT^ method to evaluate gene expression, which was normalized to β-actin levels. We used a Trizol kit extract RNA from the zebrafish. For cDNA synthesis, we used the Evo M-MLV RT Master Mix with the total RNA that had been extracted. Reverse transcription was initiated for 15 min at 37°C, after which the enzyme was deactivated for 5 s at 85°C, and it was then stored at 4°C (Zhao et al., 2024). Table 1 outlines the primers designed for specific gene targets.

**TABLE 1:** Primers sequence analysis for qRT-PCR.

Primer name	Oligo	Primer sequence	Product lengths (bp)
β-actin	Forward Primer Reverse Primer	GCTGACAGGATGCAGAAGGA TAGAAGCATTTGCGGTGGAC	20 20
NF-κB p65	Forward Primer Reverse Primer	GAGCCCTTTGTGCAAGAGAC TGGGATACGTCCTCCTGTTC	20 19
IκBa	Forward Primer Reverse Primer	GGTGGAAAGACTCCTGAAAGC TGTAGTTAGGGAAGGTAAGAATG	21 23
TNF-a	Forward Primer Reverse Primer	GGTGGSTDTTCAAAGTCGGGTGTA TGTGAGTCTCAGCACACTTCCSTD	24 24
IL-1β	Forward Primer Reverse Primer	CTCAGCCTGTGTGTTTGGGA GGGACATTTGACGGACTCG	20 19
IL-6	Forward Primer Reverse Primer	ACGACSTDAAACACAGCACC TCGSTDSTDACGCTGGAGAA	20 20

### 2.15 Statistical analysis

We used GraphPad Prism 10.0 software and FigDraw for all statistical graphs, and we used SPSS 25.0 software to test the experimental results. We used one-way analysis of variance (ANOVA) to conduct the statistical analysis and Tukey’s *post hoc* test to evaluate significant differences among experimental samples. Data from three or more independent experiments are denoted as mean ± standard error of mean (SEM). A *p*-value of <0.05 was considered to be statistically significant (*p* < 0.05 = *, *p* < 0.01 = **, *p* < 0.001 = ***) (Lee et al., 2023).

## 3 Results

### 3.1 Effect of STD on RAW 264.7 cell viability

**FIGURE 1:**
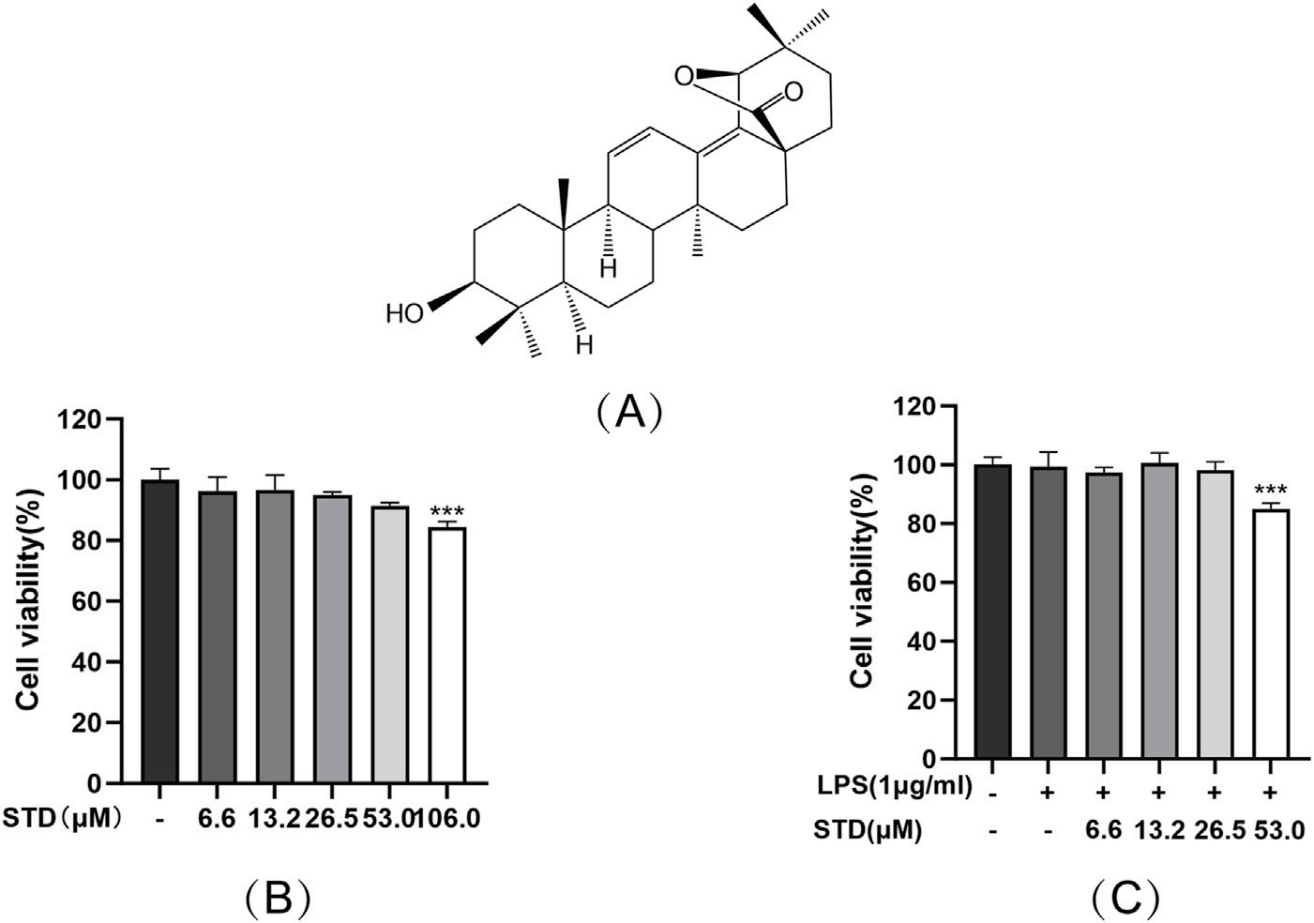
**(A)** STD chemical structure and its effect on cell viability **(B)** without LPS and **(C)** with LPS. RAW 264.7 cells were exposed to STD for 24 h at concentrations of 6.6–106.0 μM. **(B)** We utilized CCK8 assay to assess their viability. The cells were pretreated with 6.6–53.0 μM of STD for 1 h, and then LPS treatment at 1 μg/mL for 24 h. **(C)** We utilized the CCK8 assay to measure viability. Data for three or more separate experiments are given as average ± SEM values. ***P < 0.001 *versus* the blank group by one-way ANOVA with Tukey’s test.

Figure 1A illustrates the chemical structure of stytontriterpene D, which was found to be nontoxic to RAW 264.7 cells at concentrations ranging from 0 to 53.0 μM. At 106.0 μM. However, the cell viability dropped to 84.4%, exhibiting a statistically significant difference, as depicted in Figure 1B. We then assessed the cytotoxic effects of STD on LPS-stimulated RAW 264.7 macrophages. The findings indicated that concentrations of 0–26.5 μM did not significantly impair cell viability. At 53.0 μM, the survival rate decreased to 85.07%, as shown in Figure 1C. Consequently, we selected concentrations of 6.6, 13.2, and 26.5 μM as the low, medium, and high dosage groups for further experiments.

### 3.2 Effect of STD on NO and ROS in RAW 264.7 cells induced by LPS

**FIGURE 2:**
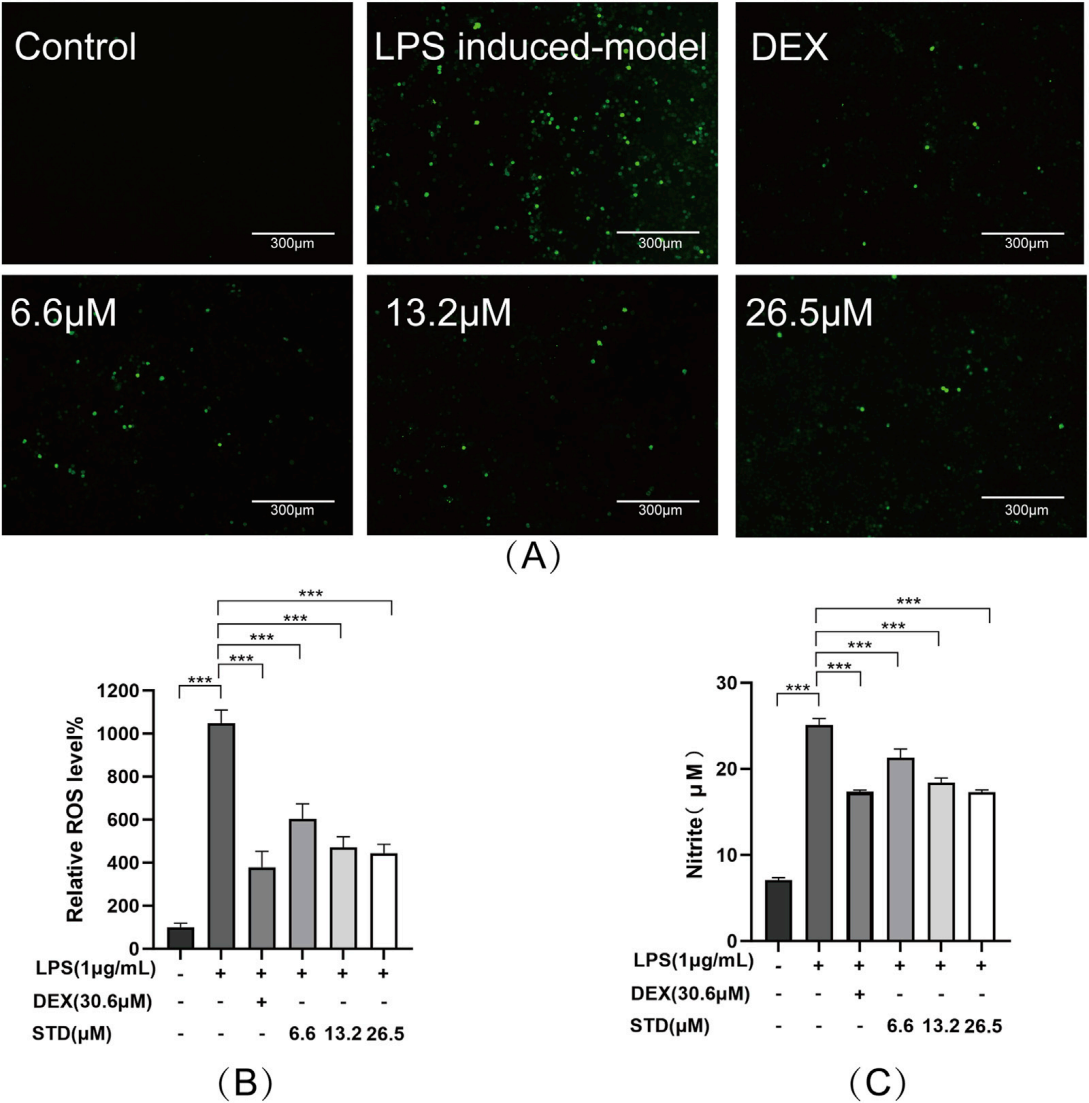
STD affected ROS and NO during inflammation induced by LPS. RAW 264.7 cells were seeded at 2 × 10^5^ per well and pretreated with different amounts of STD (6.6, 13.2, and 26.5 μM) and DEX for 1 h. Then we exposed the cells to LPS (1 μg/mL) for 24 h. We utilized DCFH-DA probe to measure levels of ROS. **(A)** We utilized a 10× fluorescent microscope to observe results. **(B)** Fluorescence intensity was quantified utilizing ImageJ software. **(C)** STD’s effect on NO released in RAW 264.7 macrophages stimulated by LPS. Data for three or more separate experiments are given as average ± SEM values. ***P < 0.001 *versus* the LPS induced-model group by one-way ANOVA with Tukey’s test.

We determined the anti-inflammatory effectiveness of stytontriterpene D by evaluating its impact on LPS-induced ROS and NO generation utilizing a DCFH-DA probe and Griess reagent, respectively. The fluorescence intensity in RAW 264.7 cells stimulated with LPS decreased as the concentration of STD increased (Figure 2B). We observed the lowest ROS levels in RAW 264.7 cells at 26.2 μM. This result was similar to that in the positive control group, which had 57.57% and 63.91% inhibition rates (Figure 2C). The release of NO in RAW 264.7 cells was approximately fourfold higher in the LPS induced-model group than in the blank group (P < 0.001). STD significantly reduced NO production induced by LPS in a dose-dependent manner (P < 0.001) compare to the LPS induced-model group. We observed an inhibitory effect of STD at 26.2 μM that was similar to the effect in the positive control, which had 31.02% and 30.82% inhibition rates. STD was able to decrease ROS and NO production induced by LPS in a dose-dependent manner.

### 3.3 Effect of STD on cytokine production in RAW 264.7 cells induced by LPS

**FIGURE 3:**
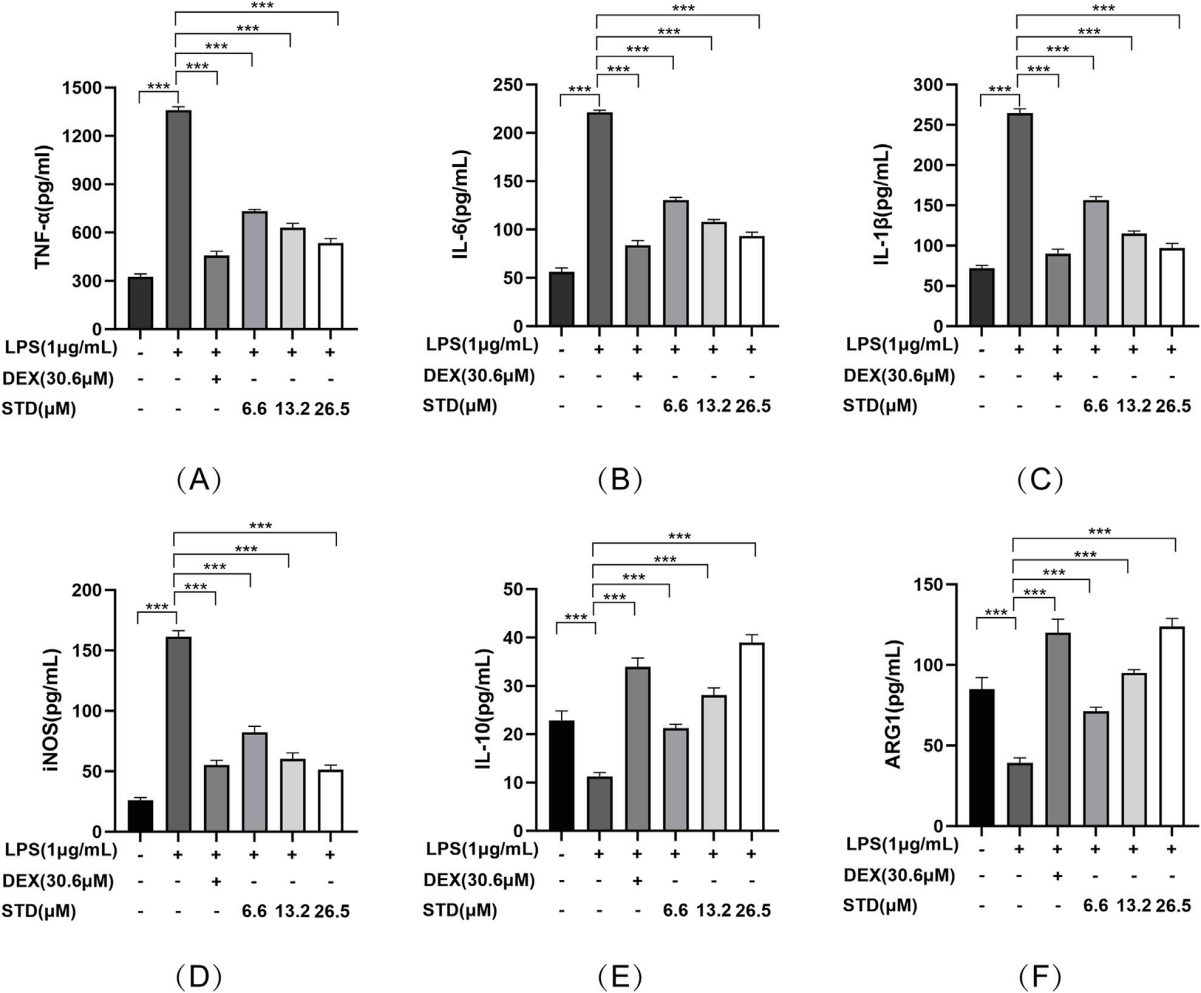
The anti-inflammatory effect of STD was determined by ELISA. RAW 264.7 cells were seeded at 2 × 10^5^ per well and pretreated with 6.6, 13.2, and 26.5 μM STD with DEX for 1 h. The cells were stimulated with 1 μg/mL LPS for 24 h. We used ELISA to quantify the levels of **(A)** TNF-α, **(B)** IL-6, **(C)** IL-1β, **(D)** iNOS, **(E)** IL-10, and **(F)** ARG1. Data for three or more separate experiments are given as average ± SEM values. ***P < 0.001 *versus* the LPS induced-model group by one-way ANOVA with Tukey’s test.

We investigated the impact of stytontriterpene D on the release of pro-inflammatory cytokines, specifically IL-6, IL-1β, and TNF-α, in LPS-stimulated RAW 264.7 cells. To elucidate the mechanism through which STD reduces LPS-induced NO production, we explored its effect on LPS-induced iNOS expression. Additionally, we examined whether STD’s mechanism involves promoting phenotype transition in RAW264.7 cells by measuring the expression levels of M2 macrophage markers IL-10 and Arg-1. We utilized ELISA to detect the level of above indicators. Following LPS stimulation, the pro-inflammatory cytokines IL-6, IL-1β, and TNF-α, along with the secretions of RAW 264.7 cells in the LPS induced-model group, were significantly elevated compared to the blank group, with increases of 4.18, 3.93, and 3.67 times, respectively. The inhibition rates of TNF-α release in the positive control group and the low, medium, and high STD groups indicated that STD effectively reduced inflammatory factors in a dose-dependent manner during the cell inflammatory response induced by LPS. Upon stimulation with LPS, the iNOS level in the LPS induced-model group was 6.18 times higher than in the blank group. After treatment with STD, the iNOS level showed a concentration dependent decrease, which was consistent with the results of the NO experiment. This suggests that the mechanism by which STD reduces NO may be related to the inhibition of iNOS expression. Furthermore, we explored STD’s regulatory effect on cell polarization. The results presented in Figure 3 demonstrate that STD suppressed the upregulation of M1 phenotype cytokines (IL-6, IL-1β, TNF-α, and iNOS) expression induced by LPS, while simultaneously inhibiting the downregulation of M2 phenotype cytokines (IL-10 and Arg-1) expression caused by LPS.

### 3.4 Effect of STD on NF-kB signaling pathway in RAW 264.7 cells induced by LPS

**FIGURE 4:**
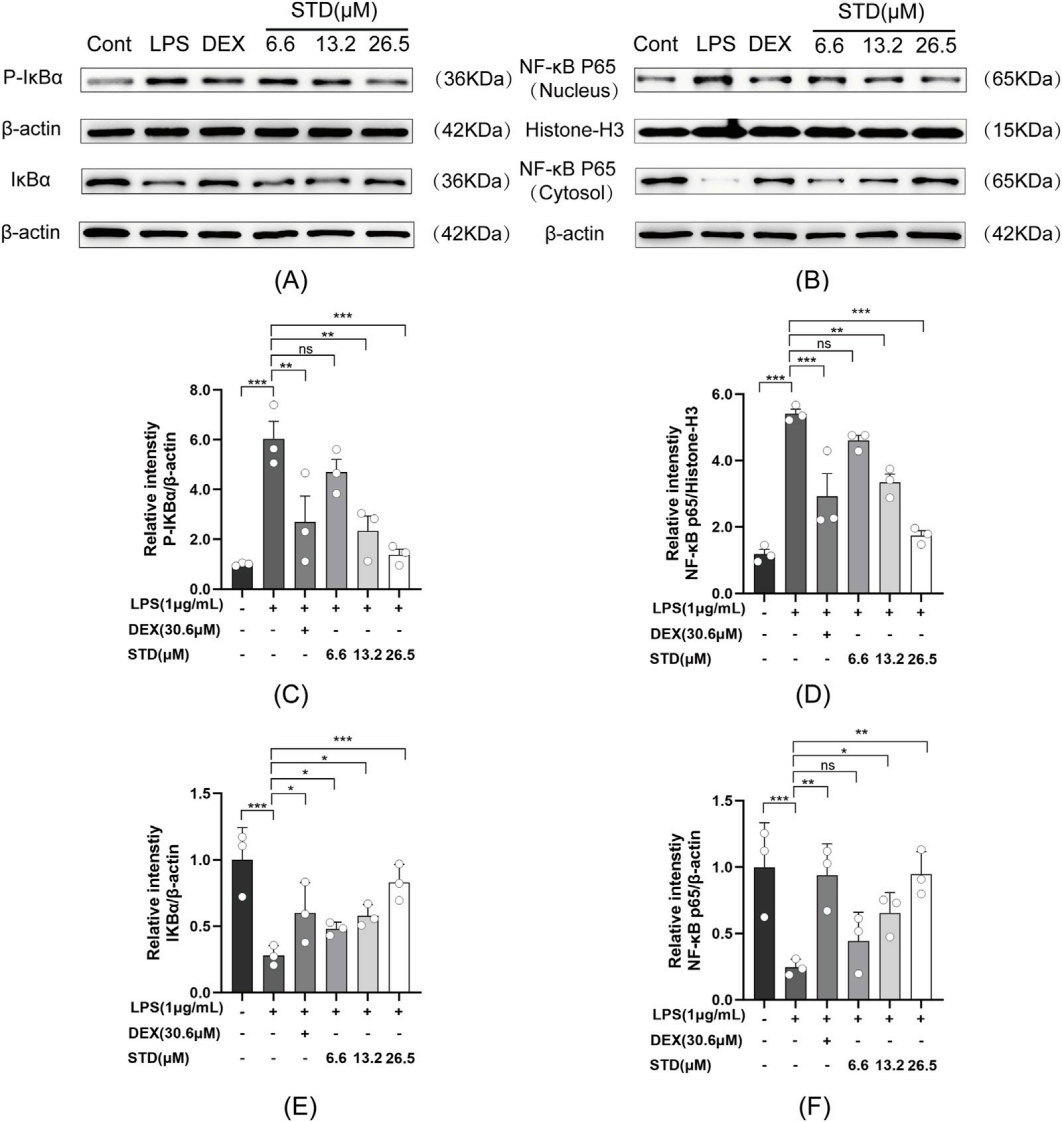
Effect of STD on **(A)** IκBα phosphorylation and **(B)** NF-κB p65 nuclear translocation. RAW 264.7 cells were seeded at 2 × 10^5^ per well and pretreated with 6.6, 13.2, and 26.5 μM STD with DEX for 1 h. We utilized ImageJ software to quantify **(C)** P-IκBα and **(E)** IκBα, which was normalized to β-actin. We also utilized ImageJ software to assess **(D)** nuclear and **(F)** cytosolic levels of NF-κB p65, which was normalized to Histone-H3 and β-actin, respectively. Data for three or more separate experiments are given as average ± SEM values. ***P < 0.001 *versus* the LPS induced-model group by one-way ANOVA with Tukey’s test.

We used western blot analysis to investigate the effect of stytontriterpene D on the NF-κB signaling pathway in RAW 264.7 cells induced by LPS. Anti-inflammatory drugs block IκBα phosphorylation and degradation to inhibit NF-κB activation (Kang and Hyun, 2020). Compared with the blank group, P-IκBα levels significantly increased and IκBα levels decreased after LPS treatment (Figure 4A). STD significantly reduced degradation and phosphorylation of IκBα induced by LPS in a dose-dependent manner. We also assessed P65 levels in both the cytoplasm and nucleus to verify the inhibition of NF-κB p65 nuclear translocation by STD (Figure 4B). A significant increase in nuclear P65 and a decrease in cytoplasmic P65 levels followed LPS treatment compared with the blank group. STD significantly blocked P65 translocation to the nucleus induced by LPS in a dose-dependent manner. STD had a superior inhibitory effect on the NF-κB pathway at a concentration of 26.5 μM relative to the positive control group.

### 3.5 Effect of STD on the survival rate of zebrafish larvae

**FIGURE 5:**
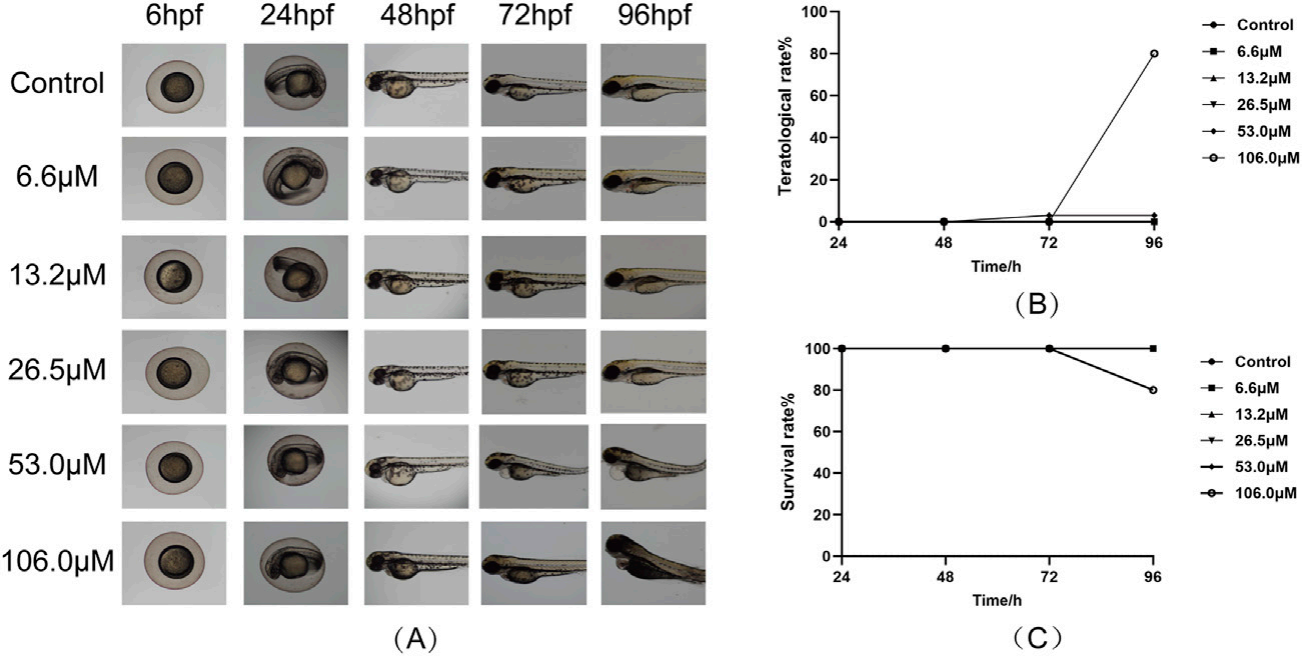
Effect of STD on the larval survival of zebrafish (n = 10). Deformity and survival rates were evaluated at 96 hpf after treatment with STD at concentrations of 6.6, 13.2, 26.5, 53.0 and 106.0 μM. **(A)** The developmental morphology at ×4 magnification for zebrafish subjected to varying STD levels; **(B)** curves of the aberration rates for these conditions; and **(C)** survival curves for zebrafish at different concentrations of STD.

To evaluate the toxicity of stytontriterpene D, we assessed larvae survival rates (Figure 5A). We did not observe any notable differences in malformation rates at concentrations of 6.6–106.0 μM compared with the control group. At 106.0 μM, however, zebrafish exhibited teratogenic effects, including pericardial edema (Figure 5B). By 96 hpf, zebrafish exposed to 106.0 μM had died (Figure 5C). These findings did not indicate any significant differences in growth morphology or mortality of zebrafish embryos at STD concentrations from 6.6 to 53.0 μM compared with the control group. Therefore, we used 13.2, 26.5, and 53.0 μM as the low, medium, and high concentrations for subsequent experiments.

### 3.6 Effect of STD on the aggregation of neutrophils and macrophages in tail-transection zebrafish

**FIGURE 6:**
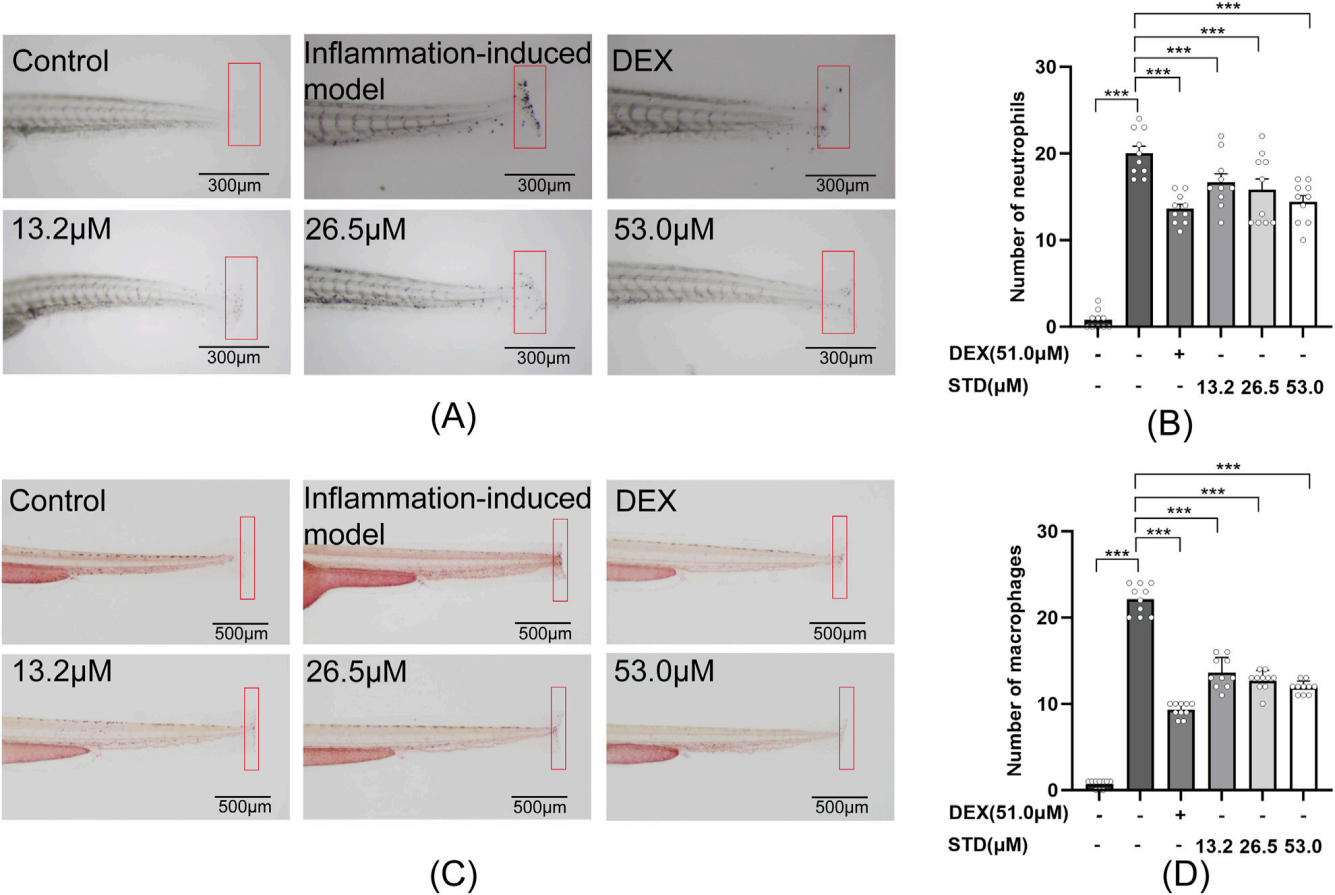
Effect of STD on neutrophils and macrophages recruitment in zebrafish after tail transection (n = 10). **(A)** The count of neutrophils in the zebrafish tail at ×40 magnification, **(B)** neutrophil numbers in the zebrafish tail, **(C)** macrophage recruitment in the zebrafish tail at ×30 magnification, and **(D)** the quantity of macrophages in the zebrafish tail. Data are shown as average + SEM values of at least three separate experiments. ***P < 0.001 versus the inflammation-induced model group by one-way ANOVA with Tukey's test.

In this study, we created a local inflammation-induced model by cutting the caudal fin of zebrafish. Sudan black and neutral red-staining techniques were employed to examine the effect of stytontriterpene D on neutrophil and macrophage aggregation in the zebrafish tail (Figures 6A,C). According to Figures 6B,D, the quantities of neutrophils and macrophages rose by 25-fold and 22.1-fold, respectively, compared with the blank group. In comparison with the inflammation-induced model, the positive control and low, medium, and high STD concentration groups showed inhibitory effects on neutrophil recruitment of 32%, 16.5%, 21%, and 28%, respectively, with significant reductions in numbers (P < 0.001). The high STD concentration group’s inhibitory effect on neutrophil recruitment was comparable to that of the positive group. Similarly, for macrophage recruitment, the positive group and low, medium, and high STD concentrations resulted in reductions of 57.9%, 38.5%, 42.5%, and 46.2%, respectively, all showing significant decreases (P < 0.001). These results indicated that STD reduced the aggregation of neutrophils and macrophages at the injury site in a dose-dependent manner.

### 3.7 Effect of STD on the aggregation of neutrophils and macrophages in copper sulfate–induced zebrafish

**FIGURE 7:**
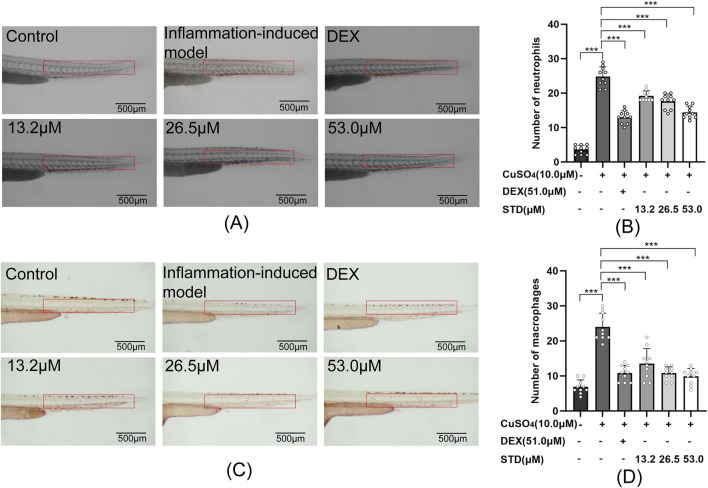
Effect of STD on neutrophils and macrophages in zebrafish induced by copper sulfides (n = 10). **(A)** Neutrophil recruitment in the lateral line of zebrafish at ×30 magnification, **(B)** the number of neutrophils in the zebrafish tail, **(C)** macrophage recruitment in the lateral line at ×30 magnification, and **(D)** the quantity of macrophages in the zebrafish tail. Data are shown as average ± SEM values of at least three separate experiments. ***P < 0.001 versus the inflammation-induced model group by one-way ANOVA with Tukey's test.

In this study, we assessed the anti-inflammatory properties of stytontriterpene D using copper sulfate in zebrafish, as depicted in Figures 7A,C. Copper sulfate induction led to a significant recruitment of neutrophils and macrophages into zebrafish. As illustrated in Figures 7B,D, there was a 6.89-fold and 3.47-fold increase in neutrophils and macrophages, respectively, over the blank group. Compared with the inflammation-induced model group, the positive control and the low, medium, and high STD concentration groups showed reductions in neutrophil recruitment by 47.5%, 22.6%, 25.8%, and 41.9%, respectively, with significant decreases (P < 0.001). Notably, the high STD concentration group showed more effective inhibition of neutrophil recruitment than the positive control. For macrophage recruitment, the positive control and the low, medium, and high STD concentration groups resulted in reductions of 55.0%, 43.8%, 55.0%, and 58.8%, respectively, also with significant decreases (P < 0.001). The high concentration of STD demonstrated superior inhibition of neutrophil recruitment compared with the positive group. These findings suggested that STD suppressed the recruitment of neutrophils and macrophages in zebrafish in a dose-dependent manner.

### 3.8 Effect of STD on copper sulfate–induced NF-kB signaling pathway in zebrafish

**FIGURE 8:**
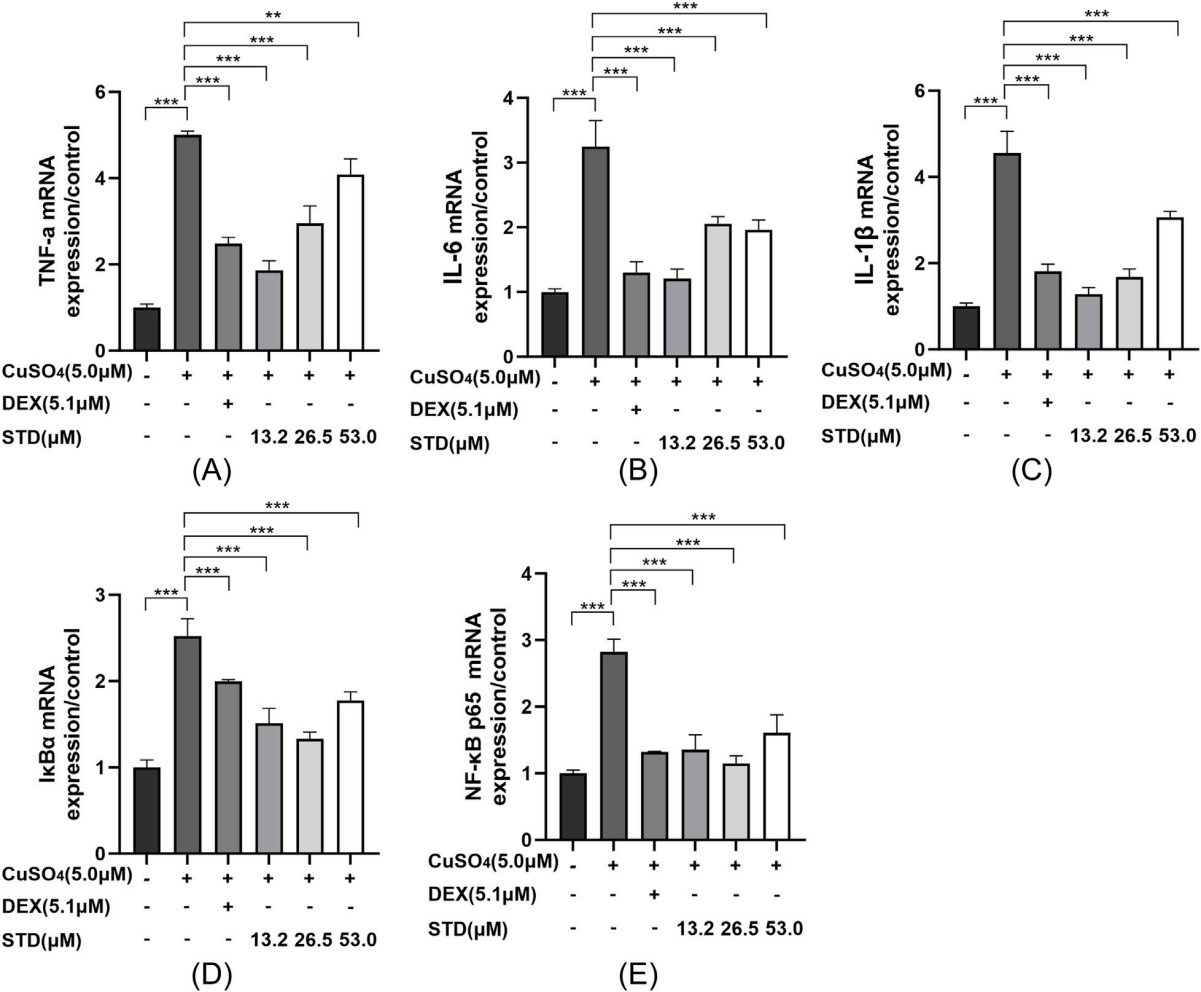
Effect of STD on the expression of inflammation-related genes in copper sulfate–stimulated zebrafish. Groups other than the control were treated with a copper sulfate solution that had 5.1 μM DEX and various amounts of STD (13.2, 26.5, and 53.0 μM). STD was able to suppress the mRNA expression of **(A)** TNF-α, **(B)** IL-1β, **(C)** IL-6, **(D)** IκBα, and **(E)** NF-κB p65 in copper sulfate–stimulated zebrafish. Data for three or more separate experiments are given as average ± SEM values of at least three separate experiments. ***P < 0.001 versus the inflammation-induced model group by one-way ANOVA with Tukey's test.

To detect the mRNA expression levels of stytontriterpene D on copper sulfate–stimulated zebrafish inflammatory cytokines IL-1β, IL-6, and TNF-α, we utilized qRT-PCR. The expression levels of IL-1β, IL-6, and TNF-α were significantly reduced by STD (Figures 8A–C). The inflammatory response was induced by the NF-κB pathway. We explored the involvement of NF-κB in STD’s anti-inflammatory effect *in vivo*. We detected STD’s effect on the mRNA expression levels of IκBα and NF-κB p65 in copper sulfate–stimulated zebrafish utilizing qRT-PCR (Figures 8D,E). The expression of IκBα and NF-κB p65 in copper sulfate–stimulated zebrafish increased 2.5-fold and 2.8-fold compared to the blank group, respectively. The upregulation of IκBα and NF-κB p65 genes in copper sulfate–stimulated zebrafish was inhibited significantly in each STD concentration group. This result demonstrated that the STD’s anti-inflammatory activity was ameliorated in copper sulfate–stimulated zebrafish by inhibiting the NF-κB signaling pathway.

## 4 Discussion

Our prior research indicated that stytontriterpene D notably decreases pro-inflammatory cytokine levels and exhibits anti-vasculitis properties (Jiang et al., 2024). Therefore, given the hypothesis that STD possesses substantial anti-inflammatory capabilities, we conducted an exploratory study on its anti-inflammatory potential, utilizing RAW 264.7 macrophage and zebrafish models for the inaugural time.

LPS, a bacterial endotoxin present in the cell walls of Gram-negative bacteria, is frequently employed to trigger immune responses in both *in vivo* and *in vitro* settings (Ulmer et al., 2000). RAW 264.7 macrophages serve as widely used cell models for investigating the immunomodulatory effects of various compounds. In the body, macrophages function as immune cells; directly respond to foreign substances; and participate in the initiation, development, and regression of inflammation. When macrophages are exposed to the bacterial cell wall component LPS, they cause inflammation (Han et al., 2019; Xu et al., 2022). Therefore, LPS is commonly utilized as a stimulant for RAW 264.7 macrophages, serving as an *in vitro* model for inflammation. RAW264.7 cells induce polarization into M1 macrophages under LPS stimulation and release a range of inflammation factors, such as ROS, NO, iNOS and cytokines, which significantly contribute to the advance and augmentation of inflammation through synergistic interactions with other inflammatory mediators (Cho et al., 2021). ROS is a natural product of cellular metabolism in the body, playing a pivotal role in various physiological processes. It has the function of promoting wound healing and repair as well as of enhancing macrophages immunity (Ouyang et al., 2022). Excessive release of ROS can disrupt redox balance within the body, leading to physiological processes, such as apoptosis, necrosis, and autophagy, which in turn exacerbate inflammation (Zhang et al., 2021; Bryan et al., 2012). To verify the influence of STD on ROS production inducted by LPS in RAW 264.7 cells, we utilized a DCFH-DA fluorescent probe. We found that ROS levels in macrophages stimulated by LPS were significantly decreased in a dose-dependent manner. These results verified that by reducing the release of ROS, STD mitigated inflammatory responses. NO is a key oxidative stress mediator believed to be involved in the onset and exacerbation of inflammation. INOS is a key enzyme in NO production, and its expression produces a large amount of NO. Excess NO can produce peroxynitrite (ONOO−), leading to local tissue damage and worsening inflammation (Ishii et al., 2017; Yun et al., 1996). The expression of NO and iNOS was measured using Griess and ELISA methods, respectively. STD inhibited the increase in their expression caused by LPS stimulation in a concentration dependent manner. Therefore, the inhibition of NO production in RAW 264.7 cells induced by LPS is related to the inhibition of iNOS expression. TNF-α, a key inflammatory marker, is prominently expressed in numerous inflammatory conditions. Therefore, it is essential in the inflammatory cascade because it can trigger inflammatory factors and mediate immune responses, and thus can damage apoptosis and tissue. The expression of TNF-α is believed to correlate directly with inflammation severity. IL-6 and IL-1β are important mediators in acute and chronic inflammation. These cytokines play pro-inflammatory and anti-inflammatory roles, promoting immune defense responses at normal levels but causing inflammatory damage at elevated concentrations. Meanwhile, these three pro-inflammatory cytokines are also highly expressed in M1 macrophages (Chen et al., 2015). LPS exposure in RAW 264.7 cells significantly increased the release of IL-6, IL-1β, and TNF-α, according to the ELISA results. Conversely, STD significantly decreased cytokine production in a dose-dependent manner. We utilized qRT-PCR in a copper sulfate–induced zebrafish to verify these results. The inflammation model demonstrated that the mRNA expression of IL-6, IL-1β, and TNF-α was decreased by STD. This result, which aligned with the ELISA findings, verified STD’s anti-inflammatory benefits at the transcriptional level. IL-10 is an anti-inflammatory cytokine that has an inhibitory effect on M1 type cells, reducing the secretion of pro-inflammatory cytokines by M1 macrophages and suppressing excessive inflammatory responses. IL-10 is also one of the important cytokines that induce macrophage polarization towards M2, and can induce high expression of ARG1 in M2 macrophages (Li et al., 2018; Wang et al., 2019). We established a model of LPS stimulated RAW264.7 cell polarization and studied the effect of STD on macrophage phenotype transformation. We found that STD significantly reduced the expression of LPS induced M1 phenotype (IL-1β, IL-6, TNF-a, and iNOS) in a concentration dependent manner, while DE significantly increased the expression of M2 phenotype (ARG1 and IL-10) mRNA, exerting anti-inflammatory effects by modulating macrophage polarization.

Zebrafish models with caudal fin resection and copper sulfate stimulation, as physical and chemical *in vivo* inflammatory models, respectively, exhibit inflammatory responses similar to those of mammals and have therefore been used to screen novel anti-inflammatory drugs (Xie Y. et al., 2021). Our research showed that STD significantly reduced the aggregation of neutrophils and macrophages in tail cut–induced and copper sulfate–induced zebrafish juvenile inflammation models. Thus, we verified the anti-inflammation activity of STD in zebrafish larvae using both physical and chemical inflammation models.

**FIGURE 9:**
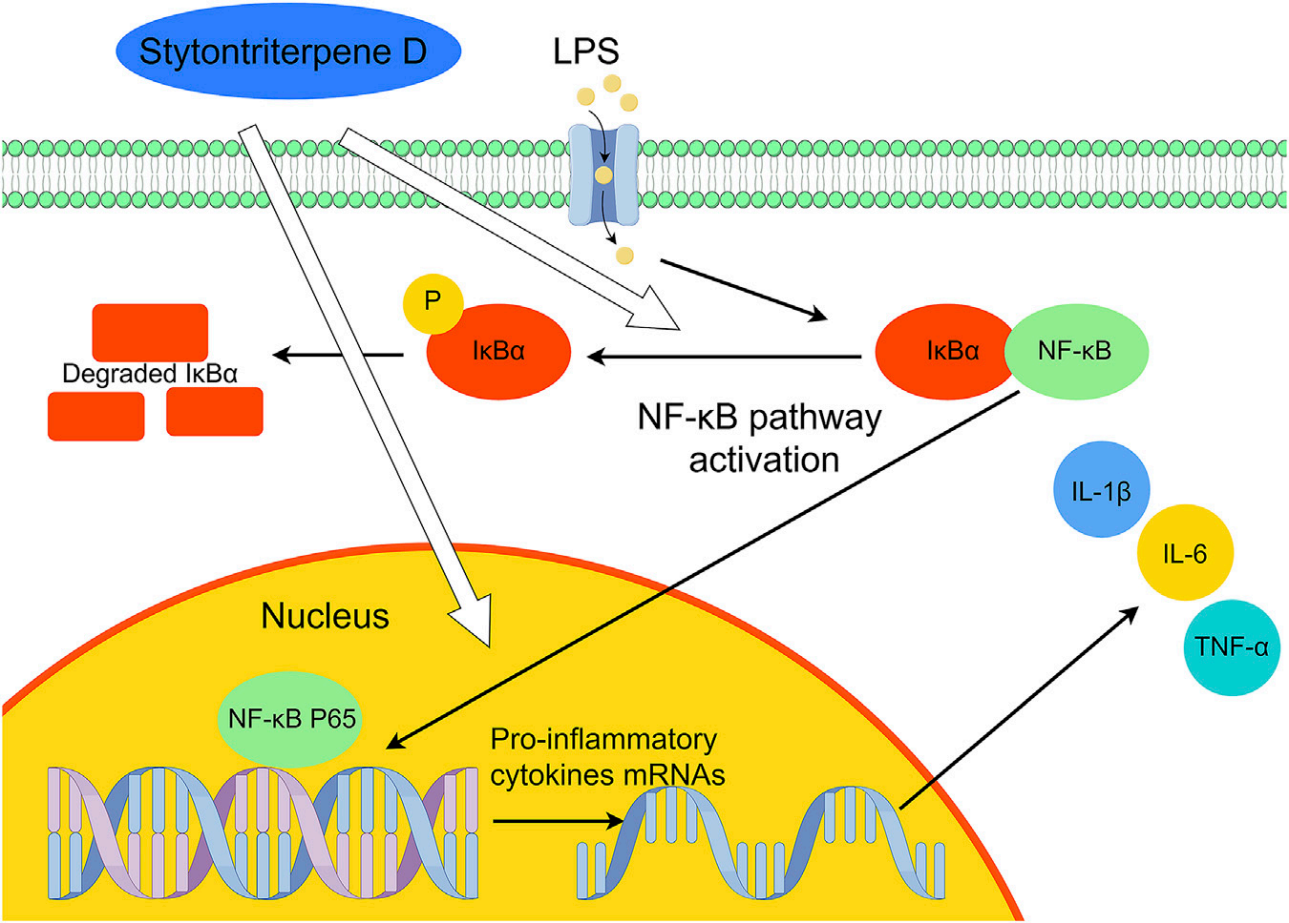
Suggested mechanism of the anti-inflammatory effects of stytontriterpene D.

Inflammation, immunity, and cancer development are tied to the NF-κB pathway. Monocytes, macrophages, and endothelial cells are activated by LPS through the NF-κB signaling pathway (Yu et al., 2020). On cell surfaces, they bind to Toll-like receptor 4, which triggers the release and synthesis of pro-inflammatory cytokines (e.g., IL-6, IL-1β, and TNF-α) (Lai et al., 2017). STD blocked the expression of IL-1β, IL-6, and TNF-α in RAW 264.7 cells stimulated by LPS at the gene transcription and protein levels. The findings of our study demonstrated that this inhibition corresponded to NF-κB pathway suppression. NF-κB is found in quiescent cells as P65-P50 heterodimers, which are bound to IκBα. Thus, they are inactively maintained in cytoplasm. After being stimulated externally by LPS, IκBα is phosphorylated and degraded, thus causing the translocation of P65 to the nucleus. Then it activates genes for chemokines and inflammatory factors (Li and Verma, 2002; Huang et al., 2013). In this study, LPS induced IκBα phosphorylation and P65 nuclear translocation in RAW 264.7 cells, according to the western blot analysis. These processes, however, were inhibited by STD. Verification in a copper sulfate–induced zebrafish model using qRT-PCR revealed that STD significantly downregulated mRNA levels of IκBα and NF-κB P65. The results of this study showed that inhibition of the NF-κB pathway caused STD to activate its anti-inflammatory effect. We confirmed these results both *in vitro* and *in vivo* (Figure 9).

## 5 Conclusion

For the first time, our findings offer evidence that the novel compound STD, extracted from *Styrax tonkinensis (Pierre) Craib ex Hartw*, exhibits anti-inflammatory properties both *in vitro* and *in vivo*. Through experimental studies on cells and zebrafish, we have observed that STD effectively inhibits inflammatory responses, with its pharmacological effects correlating to dosage. The STD may suppress inflammation by reducing the expression and phosphorylation of proteins within the NF-κB signaling pathway and regulating the phenotypic transformation of macrophages, ultimately hindering the release of various inflammatory mediators. However, in this study, we only utilized cellular and zebrafish inflammatory models. To further strengthen our findings, future experiments should incorporate mammalian models, specifically mice, to confirm the anti-inflammatory properties of STD. Additionally, while the NF-κB signaling pathway has been identified, it is imperative to investigate whether STD exerts its anti-inflammatory effects through alternative pathways as well. This comprehensive approach will aid in fully elucidating the underlying anti-inflammatory mechanisms of STD. Overall, These findings provide a theoretical basis for the anti-inflammatory effect of *S. tonkinensis extracts*, and support the potential development of STD as an anti-inflammatory drug.

## Data Availability

The raw data supporting the conclusions of this article will be made available by the authors, without undue reservation.

## Abbreviations

STD, stytontriterpene D; IL-6, interleukin-6; IL-1β, interleukin-1β; TNF-α, tumor necrosis factor-α; iNOS, inducible nitric oxide synthase; IL-10, interleukin-10; ARG1, Arginase-1; qRT-PCR, quantitative real-time PCR; LPS, lipopolysaccharide; NO, nitric Oxide; DEX, Dexamethasone; NF-κB, nuclear factor-kappa B; IκB, inhibitory iκB; ROS, reactive oxygen species; ELISA, Enzyme linked immunosorbent assay; hpf, hours post-fertilization; dpf, days post-fertilization.
