# Correction: Anti-inflammatory effect and mechanism of stytontriterpene D on RAW264.7 cells and zebrafish

**DOI:** 10.3389/fphar.2025.1620902

**Published:** 2025-10-07

**Authors:** Chuqin Yu, Gao Qiu, Xiangying Liu, Quanwei Xie, Zonghao Lin, Feng Wang, Lei Cai

**Affiliations:** ^1^ Centre for Drug Research and Development, Guangdong Pharmaceutical University, Guangzhou, China; ^2^ Guangdong Provincial Key Laboratory for Research and Evaluation of Pharmaceutical Preparations, Guangzhou, China; ^3^ Guangdong Engineering and Technology Research Center of Topical Precise Drug Delivery System, Guangdong Pharmaceutical University, Guangzhou, China; ^4^ School of Chinese Materia Medica, Guangdong Pharmaceutical University, Guangzhou, China; ^5^ Guangdong Provincial Biotechnology Research Institute (Guangdong Provincial Laboratory Animals Monitoring Center), Guangzhou, China

**Keywords:** stytontriterpene D, anti-inflammatory, mechanism, RAW 264.7 cell, zebrafish

There was a mistake in Figures 6–8 as published. An error was made in the concentrations. The corrected Figures 6–8 appear below.

**FIGURE 6:**
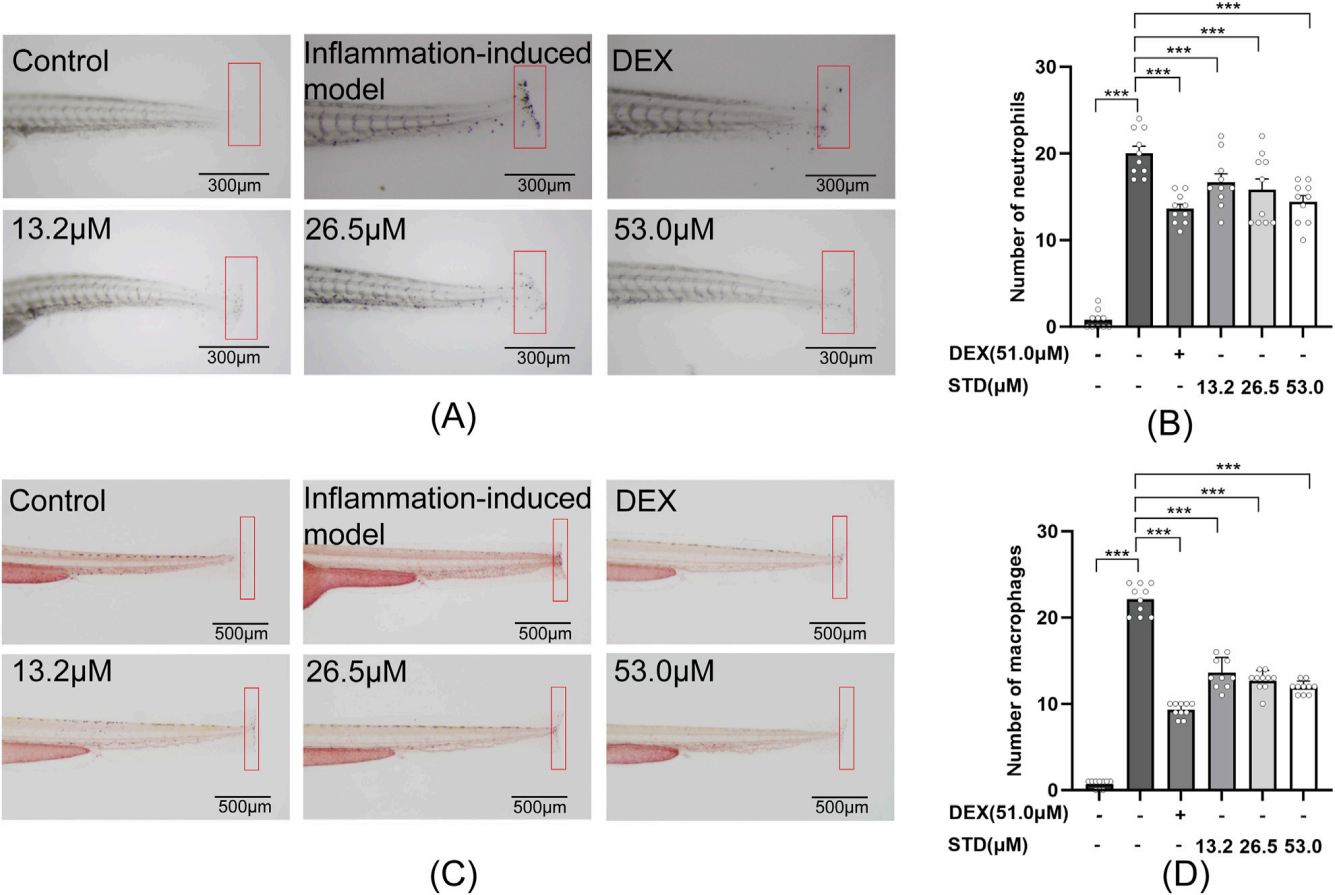
Effect of STD on neutrophils and macrophages recruitment in zebrafish after tail transection (n = 10). **(A)** The count of neutrophils in the zebrafish tail at ×40 magnification, **(B)** neutrophil numbers in the zebrafish tail, **(C)** macrophage recruitment in the zebrafish tail at ×30 magnification, and **(D)** the quantity of macrophages in the zebrafish tail. Data are shown as average ± SEM values of at least three separate experiments. ***P < 0.001 versus the inflammation-induced model group by one-way ANOVA with Tukey’s test.

**FIGURE 7:**
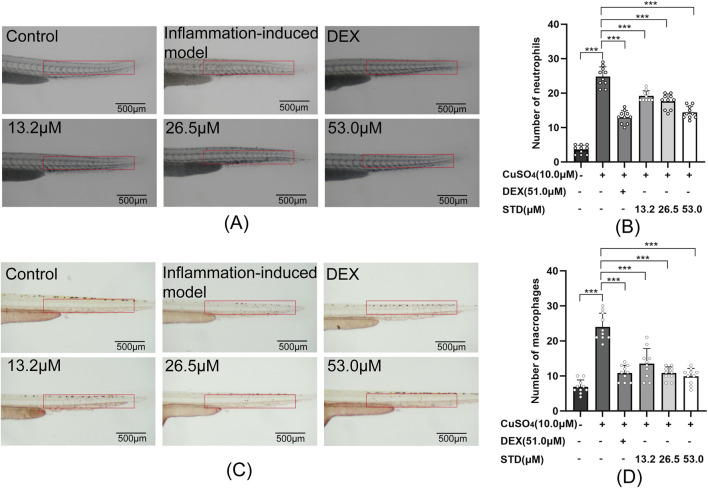
Effect of STD on neutrophils and macrophages in zebrafish induced by copper sulfides (n = 10). **(A)** Neutrophil recruitment in the lateral line of zebrafish at ×30 magnification, **(B)** the number of neutrophils in the zebrafish tail, **(C)** macrophage recruitment in the lateral line at ×30 magnification, and **(D)** the quantity of macrophages in the zebrafish tail. Data are shown as average ± SEM values of at least three separate experiments. ***P < 0.001 versus the inflammation-induced model group by one-way ANOVA with Tukey’s test.

**FIGURE 8:**
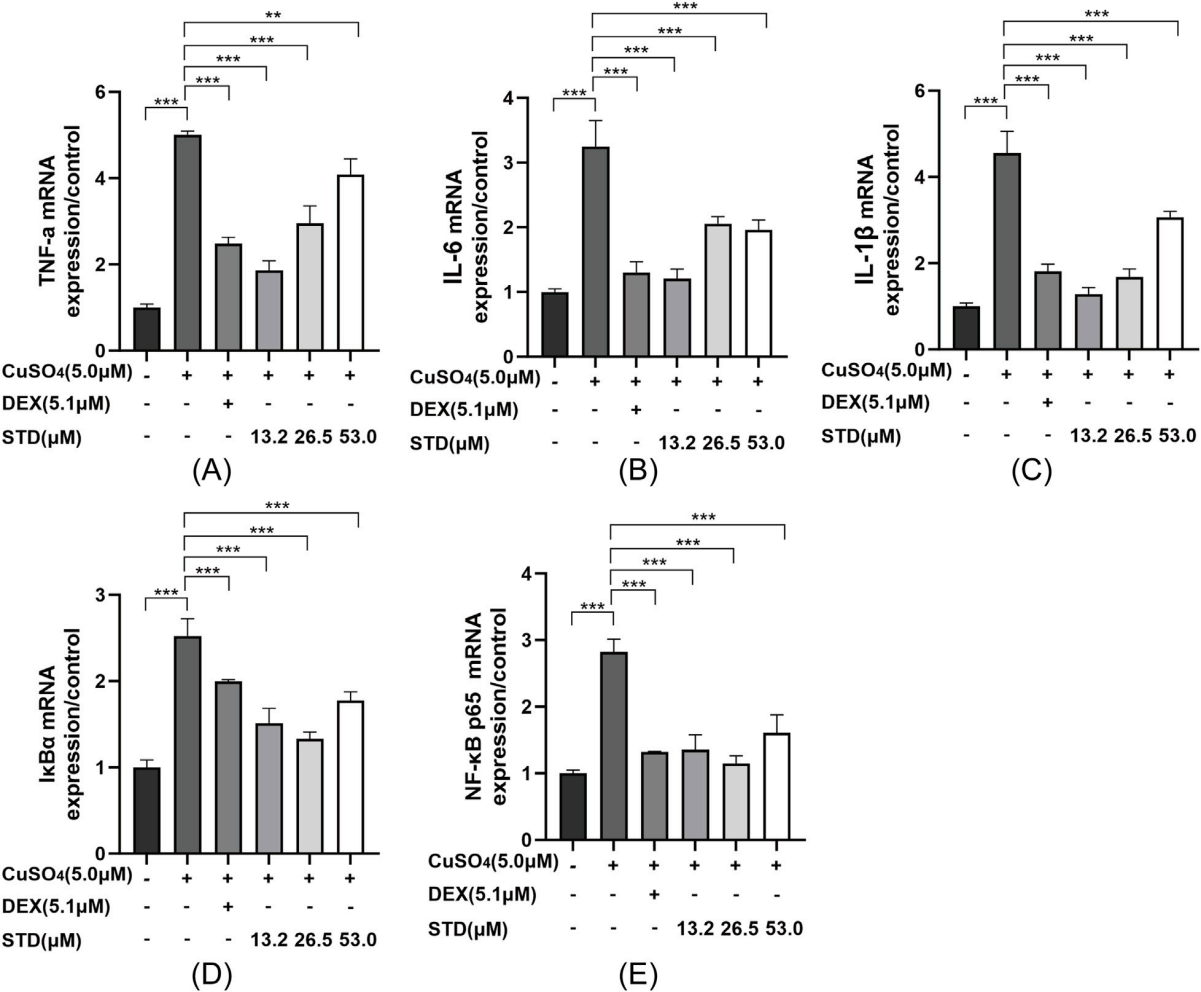
Effect of STD on the expression of inflammation-related genes in copper sulfate–stimulated zebrafish. Groups other than the control were treated with a copper sulfate solution that had 5.1 μM DEX and various amounts of STD (13.2, 26.5, and 53.0 μM). STD was able to suppress the mRNA expression of **(A)** TNF-α, **(B)** IL-1β, **(C)** IL-6, **(D)** IκBα, and **(E)** NF-κB p65 in copper sulfate–stimulated zebrafish. Data for three or more separate experiments are given as average ± SEM values of at least three separate experiments. ***P < 0.001 *versus* the inflammation-induced model group by one-way ANOVA with Tukey’s test.

There was a mistake in the caption of Figure 8 as published. It was erroneously stated that the control group were treated with a DEX solution, rather than a copper sulfate solution. As such, DEX and copper sulfate were reversed. The corrected caption of Figure 8 appears above.

TNF-α was mistakenly written as TNF-κ and NF-κB was mistakenly written as NF-αB.

A correction has been made to the section **Abstract**, Paragraph Number 2:

“**Methods:** *In vitro*, we evaluated the toxicity of STD to RAW 264.7 cells using the CCK8 method and detected the reactive oxygen species (ROS) and nitric oxide (NO) contents in cells using diacetyldichlorofluorescein (DCFH-DA) and the Griess method. We detected the levels of interleukin-6 (IL-6), interleukin-1β (IL-1β), tumor necrosis factor-α (TNF-α), inducible nitric oxide synthase (iNOS), interleukin-10 (IL-10), and arginase-1 (ARG1) via enzyme-linked immunosorbent assay and measured the expression of related proteins in the NF-κB pathway via western blotting. The toxicity of STD to AB zebrafish was detected *in vivo*, and the recruitment of neutrophils and macrophages was evaluated in tail cut-induced and copper sulfate-induced zebrafish inflammation models. We used quantitative real-time polymerase chain reaction to study the expression of inflammation-related genes in zebrafish with inflammation induced by copper sulfate.”

Throughout **Sections 2.12-2.13**, the concentration of DEX was mistakenly written as 61.2 μM, while the correct concentration is “DEX (51.0 μM)”.

All instances have been corrected to “DEX (51.0 μM)” in the following sections:

**Materials and methods**, 2.12 Tail transection–induced inflammatory model in zebrafish, sub-sections 2.12.1 and 2.12.2; and Materials and methods, 2.13 Copper sulfate–induced inflammatory model in zebrafish, sub-sections 2.13.1 and 2.13.2.

Throughout Section 2.13, the unit for copper sulfate was erroneously written as 1.6 μM, rather than “10.0 μM”. All instances have been corrected to “10.0 μM copper sulfate” in the following sections: **Materials and methods**, 2.13 Copper sulfate–induced inflammatory model in zebrafish, sub-sections 2.13.1 and 2.13.2.

STD was mistakenly written as DE.

A correction has been made to the section **Discussion**, Paragraph number 2. The corrected sentence is:

“We found that STD significantly reduced the expression of LPS induced M1 phenotype (IL-1β, IL-6, TNF-a, and iNOS) in a concentration dependent manner, while STD significantly increased the expression of M2 phenotype (ARG1 and IL-10) mRNA, exerting anti-inflammatory effects by modulating macrophage polarization.”

The original article has been updated.

